# Rheumatic Manifestations and Diseases From Immune Checkpoint Inhibitors in Cancer Immunotherapy

**DOI:** 10.3389/fmed.2021.762247

**Published:** 2021-11-04

**Authors:** Pan Shen, Xuan Deng, Zhishuo Hu, Zhe Chen, Yao Huang, Ke Wang, Kai Qin, Ying Huang, Xin Ba, Jiahui Yan, Liang Han, Shenghao Tu

**Affiliations:** ^1^Department of Integrated Traditional Chinese and Western Medicine, Tongji Hospital, Tongji Medical College of Huazhong University of Science and Technology, Wuhan, China; ^2^Department of Nephrology, Zhongnan Hospital of Wuhan University, Wuhan, China; ^3^Department of Emergency, Wuhan No.1 Hospital, Wuhan, China; ^4^Department of Integrated Traditional Chinese and Western Medicine, The Central Hospital of Wuhan, Wuhan, China

**Keywords:** rheumatic diseases, immune-related adverse events, immune checkpoint inhibitors, arthritis, myositis, vasculitis

## Abstract

Immune checkpoint inhibitors (ICIs), which can enhance antitumor immunity and inhibit cancer growth, have revolutionized the treatment of multiple cancers and dramatically decreased mortality. However, treatment with ICIs is directly associated with immune-related adverse events (irAEs) because of inflammation in off-target organs and autoimmunity resulting from non-specific immune activation. These irAEs can cause rheumatic diseases and manifestations such as inflammatory arthritis, polymyalgia rheumatica, myositis, vasculitis, Sicca and Sjogen's syndrome, and systemic lupus erythematosus. Early diagnosis and treatment of these adverse events will improve outcomes and quality of life for cancer patients. The treatment of rheumatic diseases induced by ICIs requires multidisciplinary cooperation among physicians. Furthermore, the underlying mechanisms are not fully understood and it is difficult to predict and evaluate these side effects precisely. In this review, we summarize available studies and findings about rheumatic irAEs, focusing mainly on the clinical manifestations, epidemiology, possible mechanisms, and guiding principles for treating these irAEs.

## Introduction

Cancer, one of the most serious diseases affecting human health, has become the second leading cause of death worldwide—accounting for nearly a quarter of deaths—and the number of patients and deaths is growing rapidly every year (1). Immune-checkpoint inhibitor (ICI) therapy has provided a promising treatment strategy for patients (2). ICIs can overcome self-tolerance and enhance antitumor immunity through several approaches, mainly via the action of anti-cytotoxic T lymphocytes-associated antigen 4 (CTLA-4), anti-programmed death (PD)-1, and PD-ligand (L)-1 antibodies, according to the checkpoint inhibition target (3). ICIs therapy also can cause a unique and distinct immune-driven toxicity or autoimmune side effects termed “immune-related adverse events” (irAEs), which might be due to immune stimulation or loss of self-tolerance (4). IrAEs can affect multiple organs; this varies from patient to patient, and different patients present with diverse clinical manifestations ranging from mild (grade 1), to moderate (grade 2), severe, and generally requiring hospitalization (grade 3), life-threatening (grade 4), and death caused by adverse drug reactions (grade 5) according to the Common Terminology Criteria for Adverse Events (CTCAE) system (5, 6).

In some cases, irAEs are similar to various rheumatic diseases, including Sicca and Sjogren's syndrome (SS), myositis, arthritis, vasculitis, polymyalgia rheumatica (PMR), and systemic lupus erythematosus (SLE) (7). However, some of these inflammatory symptoms may be overlooked and confused with the related symptoms during the cancer treatment by physicians. In this review, we summarize the latest studies and findings about the clinical manifestations, unresolved questions, and underlying immune mechanisms, and propose approaches to the management of rheumatic diseases resulting from the use of ICIs.

## Underlying Mechanisms and Potential Biomarkers

Most studies report clinical observations and the possible mechanisms are not fully understood (8). The incidence of rheumatic irAEs is associated with several factors, such as type of ICIs, dosage, the combination of ICIs used, and the accumulated dosage. Patients treated with an anti-CTLA-4 antibody are more vulnerable to irAEs compared with those treated using a PD-1/PD-L1 antibody (9). Different degrees of rheumatic disease have been observed in CTLA4- or PD-1-deficiency animal models (10). Autoimmune manifestations are likely to be associated with dysfunction or downregulation of CTLA4 (11). The deletion of CTLA-4 in adult mice showed an increase of T follicular helper and T follicular regulatory cells, resulted in antigen-specific expansion of antibody responses (12). ICIs target early stages of T-cell development, leading to increase in autoreactive T-cells, and autoimmune rheumatologic manifestations. When blocking PD-1, the autocrine binding of PD-1 to PD-1 ligands is inhibited, preventing apoptosis of B cells and improving the possibility for these cells to survive longer to produce specific antibodies.

In some anti-cyclic citrullinated peptide antibodies (ACPA)-positive patients with rheumatoid arthritis (RA), ICI therapy make them vulnerable to acute onset disease, especially those treated with anti-PD-1 therapeutics (13). In addition, T cell exhaustion could be correlated with disease activity in anti-nuclear cytoplasmic antibody (ANCA)-associated vasculitis and SLE (14). ICIs could induce epitope spreading, a diversification of epitope specificity of intra-tumoral T cells away from an initial focused, dominant epitope-specific immune response to a tumor (15). The increased levels of inflammatory cytokines, such as IL-17, TNF-α, are also associated with rheumatic irAEs, and targeted inhibitors can treat these adverse reactions (16, 17).

Unfortunately, it is difficult to predict the type and severity of rheumatic irAE because of the lack of no putative biomarker, classic HLA associations, perturbations of peripheral B cells, cytokines, and signature autoantibodies might provide crude estimates of the risk of irAEs (15, 18, 19).

## Autoimmune IrAEs

Recent studies report that irAEs have occurred in ~90% of patients treated with ICIs, typically in the endocrine system, gastrointestinal tract, skin, heart, and lungs; the precise site varies from patient to patient (8). The incidence rates of rheumatic irAEs are evaluated based on numerous case series and retrospective clinical trials, many of which are phenotypically similar to classic rheumatic diseases (20). Most categories of rheumatic irAEs have been reported in ICIs trials; the most common event is arthritis (21).

### Inflammatory Arthritis

Approximately 5% of the patients show clinical syndromes of arthritis or arthralgia after ICI treatment with anti-CTLA-4 (3–9%), anti-PD-1/PD-L1 (7–11%), and combination therapy (11%) (22, 23). A single-center prospective study revealed that 6.6% of patients (35/524) receiving ICIs developed rheumatic diseases (24). However, even this figure might be an underestimate because arthritis symptoms sometimes are ignored. The time between the onset of symptoms and the time of diagnosis in patients receiving ICI therapy varies. In a cohort study of 30 arthritis patients treated with ICIs (25), the median time of onset was 3 (1.3–12) months after ICI initiation, and knee arthritis was referred to a rheumatologist within one to two months, and arthritis in the small joints within 1 year. To date, three subtypes of inflammatory arthritis are characterized, including polyarthritis, which similar to RA; seronegative spondyloarthritis; and true reactive arthritis (26). Patients generally have joint pain and morning stiffness but no objective joint swelling, sometimes with accompanying tenosynovitis, enthesitis, or psoriasis (27, 28). Initially, arthritis has an additive pattern manifested as oligoarthritis, which subsequently proceeds to inflammatory polyarthritis. About two-thirds of arthritis affects the metacarpophalangeal joints, wrists, and larger joints, similar to RA (25, 29). Although joint fluid is found to show inflammation, laboratory tests do not show markers or specific findings. In the subtype of spondyloarthritis with large-joint phenotype, patients are HLA-B27 negative (30). A small number of patients are seropositive for RF and/or ACPA.

Arthritis can be detected by ultrasound, X-rays, computed tomography (CT), and magnetic resonance imaging (MRI). Studies suggest that PET-CT and CT can be helpful in detecting synovitis with a high sensitivity and specificity in patients with ICI-induced arthritis (30, 31). In MRI and musculoskeletal ultrasonography tests, imaging always shows tenosynovitis, Doppler-positive synovitis, erosive disease, and joint effusions (26, 32). In a retrospective study of MRI in ICI-induced inflammatory arthritis (33), it is common to find tenosynovitis and synovitis in hands and wrists, while osseous erosions were found only in a few patients, which might indicate a worse prognosis. In other cases, joint pain and swelling also can result from tumor progression rather than ICIs (34). The severity of ICI-induced joint symptoms can range from mild to severe, but most are usually mild or moderate, and ICI therapy can be continued in these cases (35). Patients with mild syndromes only required NSAIDs or low-dose prednisone, with severe joint pain associated with stiffness or non-traumatic joint swelling should be referred to a rheumatologist.

### PMR

A prospective study reported an estimated prevalence of 2.1% in 524 patients with ICI therapy developed a new-onset PMR-like syndrome (24). PMR syndrome is characterized by acute predominant joint pain and morning stiffness (shoulders, neck, hips, thighs) (24, 36–39). It commonly occurs about 3 months after starting ICI treatment and accompanied by other rheumatic irAEs (40). Given proximal limb involvement in ICI-associated PMR, it is important to consider myositis in the differential diagnosis. A multicenter and systematic review (40) evaluated 49 patients with ICI-induced PMR, and observed atypical features, such as the involvement of other joints (mostly knees and hands), the absence of high inflammatory indicators. In addition, it is important to seek symptoms suggesting temporal arteritis, such as headache and visual impairment, due to the same spectrum of disorders and frequently co-existence between PMR and temporal arteritis. Patients occasionally have a positive RF or ACPA. Most cases of ICI-induced PMR respond well to moderate doses of steroids, but the clinician should be aware that atypical cases with normal acute phase reactants exist and more aggressive therapy is sometimes required.

### Myositis

Brahmer et al. reported an incidence of myalgia of 2% in patients receiving nivolumab treatment (41), while a prospective study showed the incidence to be up to 18.2% (42). However, prospective studies have only reported small-scale cases of myositis induced by ICIs, indicating a possible low prevalence. Myositis is characterized by muscle weakness in the proximal limbs and muscle inflammation as observed via neurophysiological and histopathologic detection, with or without myalgia (43). ICI-induced myositis can occur within 1 month of ICI initiation, with asymptomatic plasma creatine kinase (CK) and diffuse weakness (44). In some cases, CK can be highly elevated to 70 times the normal value, and accompanied by rhabdomyolysis (45). Moreover, ICI-induced myositis is strongly associated with myocarditis (11.3%) and myasthenia (11.9%), resulting in increased mortality (46, 47). Several studies have reported the appearance of myositis-like syndromes, myasthenia, and cardiac manifestations after a few infusions of ICI therapy (45, 48, 49). In addition, paraneoplastic myositis, such as dermatomyositis, induced by cancer but not ICI treatment, is difficult to distinguish from ICI-induced myositis (50). The time of onset of myositis symptoms might aid differentiation within 1–2 months of ICI treatment.

Muscle pathology in ICI myositis showed infiltration of cytotoxic T cells, multifocal necrotic myofibers, and endomysium inflammation, consisting mainly of CD68+ PD-L1+ cells and CD8+ PD-1+ cells (44, 51). An autopsy study in ICI myositis found CD81+ T-cell in the tumor, heart, and skeletal muscle, indicating the possible association between muscle injury and cross-reactive T cells in tumor and muscles (52).

CK levels do not perfectly reflect disease severity in patients: it is preferable to evaluate myositis via the detection of the severity of muscle weakness, CK levels, and extra-skeletal muscle organ complications (53). ICI treatment should not be discontinued unless severe pain, functional impairment, true muscle weakness, and/or myasthenia features, or cardiac involvement occurs. For severe or refractory patients, hospitalization and treatment are required, including corticoid pulse therapy, intravenous immunoglobulin, biological agents, plasmapheresis, and immunosuppressants.

### Vasculitis

ICI-induced vasculitis is rare, the most frequent malignancy with vasculitis in ICI treatment is melanoma (54). Many different symptoms are observed in ICI-induced vasculitis, such as weight loss, fatigue, stomach ache, arthralgias or arthritis, fever, palpable purpura, and myalgias. In a study of anti-PD-1/PD-L1/CTLA-4 administration in cancer patients, 37.74% of them developed vasculitis, including patterns of large vasculitis (isolated aortitis, giant cell arteritis) and vasculitis of the nervous system (isolated vasculitis of the peripheral nervous system, primary angiitis of the central nervous system) (55). Isolated vasculitis can affect the retina and testicles (56, 57). Cutaneous granulomatous (58) and leukocytoclastic vasculitides (59) have also been shown in several studies. In addition, small-vessel vasculitis was also detected in patients receiving ICIs, such as eosinophilic granulomatosis with polyangiitis (60) and cryoglobulinemic vasculitis (61). Serologies rarely are positive, and the diagnosis is lack of autoantibodies in vasculitis. In a recently case with NSCLC, the patient developed acral vasculitis after receiving anti-PD-L1 mAb therapy (62). The laboratory test results revealed a positive perinuclear-ANCA, and negative anti-myeloperoxidase (MPO) and anti-proteinase 3 (PR3) antibodies. ANAs and cold agglutinins were not detected. Antiphospholipid body antibodies to IgG and IgM were negative. The detection of vasculitis only depends on imaging methods in a few cases; biopsy is required to confirm a diagnosis in some patients. Considering systemic vasculitides may result in severe organ damage, treatment of ICI may be discontinued, with the remedy therapy of hydroxychloroquine, glucocorticoid, and plasma exchange (63, 64). It should be noted that acral vasculitis with digital ischemia could be concurrent with cancer (65, 66); discontinuation of ICIs and steroid therapy might distinguish ICI-induced vasculitis and others.

### SLE

SLE is not uncommon after ICI treatment (67), and likely involves different underlying mechanisms to other autoimmune diseases. In a study of 4,870 irAE cases after ICI, the median time for the development of SLE among 18 patients was 196 days after initiation of ICI, and the mean age was 61 years (68). SLE often manifests with lupus nephritis, immunoglobin (IgG and IgM) and complement complexes (C3 and C1q) in the mesangium with hypertrophy of podocytes detected in a melanoma patient treated with ipilimumab (69). The patient had increased titers of anti-double-stranded DNA antibodies and normal level of serum complement. Withholding ipilimumab and the administration of prednisolone relieved symptoms of ICI-induced lupus nephritis.

### SS

A recent study reported that in 17 cases of SS following ICI treatment, the median age was 63 years, with median time of onset of SS after ICI initiation being 3.8 months (70). Although these percentages are not precise, the incidence of SS in ICI-treated patients has been reported to range from 1.2 to 24.2% in published studies (40, 71). ICIs-induced SS is mainly manifested as xerostomia and parotid enlargement (72). Antinuclear antibodies, anti-SSA/B antibodies, RF, and extractable nuclear antigens are absent in the majority of patients (73, 74). Salivary gland biopsies are helpful to make a diagnosis, with the characteristics of obvious lymphocyte infiltration (CD3+ and CD4+ T cells), epithelial injury, and a significant absence of B cells. Treatment of ICI-induced SS consists of saliva and tear substitutes in most cases; severe symptoms require steroids and cessation of ICI.

## ICIs in Patients With Preexisting Rheumatic Disease

Most trials of ICIs exclude tumor patients with autoimmune diseases; therefore, whether the use of ICIs will affect the progression of rheumatic diseases is not completely understood. However, the non-specific upregulation of T cell activation and the inhibition of Treg activity due to ICI treatment could promote inflammation and autoimmunity in patients with preexisting rheumatic diseases (75). In a system review of 123 patients with malignant tumors and rheumatic diseases, it was reported that 92 cases (75%) developed irAEs and/or progression and recurrence of autoimmune disease (76). Several studies have reported that ICI therapy is associated with exacerbation of underlying rheumatic disease such as RA, SLE, psoriatic arthritis, SS, Crohn's disease, polymyalgia rheumatic, and Graves' disease, in 6–43% of patients with preexisting confirmed autoimmune disease; furthermore, ~75% of rheumatic disease patients developed irAEs after the therapy of ICIs (77–80). The exacerbation of rheumatic disease is related to the type of malignant tumors and previous treatment. At present, although preexisting rheumatic diseases are not considered be an absolute contraindication to ICIs, they represent a major obstacle to immunotherapy for patients with malignant tumors. In addition, the progression of rheumatic diseases has been reported frequently in patients with active disease symptoms rather than remission during ICI administration. Therefore, it is important to perform careful assessment before the initiation of ICI therapy because of the potential risk. Even if the risk of disease progression is higher in these patients, there is no reason to exclude them from receiving ICI treatment, especially considering rheumatic disease itself is not life-threatening (28).

## Management and Remedy Strategies for Rheumatic irAEs

No definitive prospective trials and official guidelines have been conducted and treatment recommendations for rheumatic irAEs are thus lacking. In general, treatment approaches for rheumatic irAEs should be selected after discussion between experts from oncologists and rheumatologists, for optimal therapy (81). It is of the utmost importance to facilitate early diagnosis and treatment in the specific management for rheumatic irAEs in order to prevent permanent damage (82, 83). The general treatment principle suggests managing autoimmune irAEs depending on the severity of symptoms according to the CTCAE system (84) (Table 1). For grade 1, analgesics (such as NSAIDs) or local injections with steroids could be used to alleviate symptoms; systemic corticosteroids are generally not required and treatment with ICIs can continue with close monitoring. In case of grade 2 symptoms, ICI therapy may be temporarily withheld until symptoms are resolved or improve to grade 1 or less. Corticosteroids are generally needed; some of the disease-modifying antirheumatic drugs (DMARDs) such as methotrexate, hydroxychloroquine, and sulfasalazine could also be used to manage irAEs in order to avoid side effects related to corticosteroids. In patients with grade 3 or higher events, ICI therapy should be discontinued, and high doses of steroids could be used with a gradual tapering course when symptoms alleviate to grade 1 or less (85). When steroids are not effective, or patients are unable to taper steroids, biological agents (TNF or IL-6 inhibitors) and DMARDS (such as hydroxychloroquine, methotrexate, and sulfasalazine) could be used as second-line therapy recommendations (86, 87). The early administration of these agents is associated with faster resolution of symptoms and a decreased infection risk compared with high-dose steroids alone (88–90). A retrospective study has suggested patients treated with ipilimumab can safely switch to PD-1 inhibitors if they have serious adverse reactions, and only 3% of people have serious adverse reactions again. This may indicate that a specific pathway, rather than systemic immunity, is involved in the occurrence of immunotoxicity (91). In future studies, rheumatic irAEs could be integrated into the grading system of non-irAEs induced by ICIs to improve management and facilitate decisions regarding decreasing ICIs and discontinuing therapy. Investigation of potential long-term benefits to patients requires more multi-center, large-scale prospective studies (92).

**Table 1:** Manifestations and management of selected rheumatic irAEs.

**Rheumatic irAEs**	**Differences from classic rheumatic disease**	**Testing**	**Treatment**
Arthritis	1. Can manifest as mono-, oligo- or polyarthritis 2. Myofasciitis may be prominent early in the course of disease 3. RF and CCP are often negative 4. DMARDs are needed when relapse occurs during steroid tapering	ANA, CCP, RFESR, CRP	Grade 1: NSAIDs, intra-articular steroid injection. Continue ICI therapy. Grade 2: NSAIDs, intra-articular steroid therapy, low-dose corticosteroids. Temporal discontinuation of ICI therapy. Grade 3–5: NSAIDs, intra-articular steroid therapy, prednisone (1–1.5 mg/kg). TNF/IL-6 inhibitors, hydroxychloroquine, sulfasalazine, methotrexate can be used if unable to taper steroids. ICI cessation.
PMR	1. Some patients are not responsive to low-dose prednisone, higher doses of steroids may be needed and patients always have not increased inflammatory markers 2. Involvement of joints, such as the knees and hand joints	RF, ESR, CRP, CCP	Grade 1: NSAIDs, subacromial bursa injection. Prednisone (0.1–0.5 mg/kg). Hydroxychloroquine, sulfasalazine, methotrexate can be used if unable to taper steroids. Continue ICI therapy. Grade 2: NSAIDs. Prednisone (0.5–0.8 mg/kg). If unable to taper steroids, consider methotrexate and IL-6 inhibitors. Temporarily discontinue ICI therapy. Grade 3–5: NSAIDs. prednisone (1–1.5 mg/kg). Methotrexate, IL-6 inhibitors, ICI cessation.
Myositis	1. Autoantibodies are usually absent 2. High-dose steroids are usually required 3. Increased frequency of concurrent myasthenia and/or cardiac involvement 4. Can manifest as myalgia and oculomotor symptoms	CK, EMG, MRI, muscle biopsy troponin, transaminases, ESR, CRP, anti-striatedMuscle, acetylcholine receptor, and myositisAntibody panel Echocardiogram and EKG to screen for concomitant myocarditis	Grade 1: NSAIDs. Prednisone 10–20 mg daily. Continue ICI therapy. Grade 2: Prednisone (0.5–l mg/kg). Temporarily discontinue ICI therapy. Grade 3–5: Methylprednisolone, methotrexate, azathioprine, mycophenolate mofetil, plasma change. Discontinue ICI therapy.
Vasculitis	Inflammatory markers are commonly increased, but autoantibodies are rare	RF, ESR, CRP, CCP, ANCA	Grade 1: - Grade 2: prednisone (initially 1 mg/kg), hydroxychloroquine, methotrexate. Temporarily discontinue ICI therapy. Grade 3–5: rituximab or plasma exchange. Discontinue ICI therapy.
SS	1. Dry mouth is the most prominent symptom 2. Autoantibodies, including anti-Ro and anti-La antibodies, are rare 3. Rare parotitis	ANA, RF, ESR, CRP, anti-Ro, anti-La antibodies	Grade 1: Biotin, cevimeline or pilocarpine. Continue ICI therapy. Grade 2: Prednisone (0.1–0.5 mg/kg). Temporarily discontinue ICI therapy. Grade 3–5: Prednisone (1–1.5 mg/kg). Discontinue ICI therapy.
SLE	1. Patients are always older 2. No striking female predominance 3. Autoantibodies are usually absent	ANA, anti-dsDNA antibodies, ESR, CRP, C3, C4.	Grade 1: NSAIDs, hydroxychloroquine. Continue ICI therapy. Grade 2: Prednisone (0.1–0.5 mg/kg). Temporarily discontinue ICI therapy. Grade 3–5: Mycophenolate mofetil, prednisone (1–1.5 mg/kg). Discontinue ICI therapy.

## Future Directions

**Figure 1:**
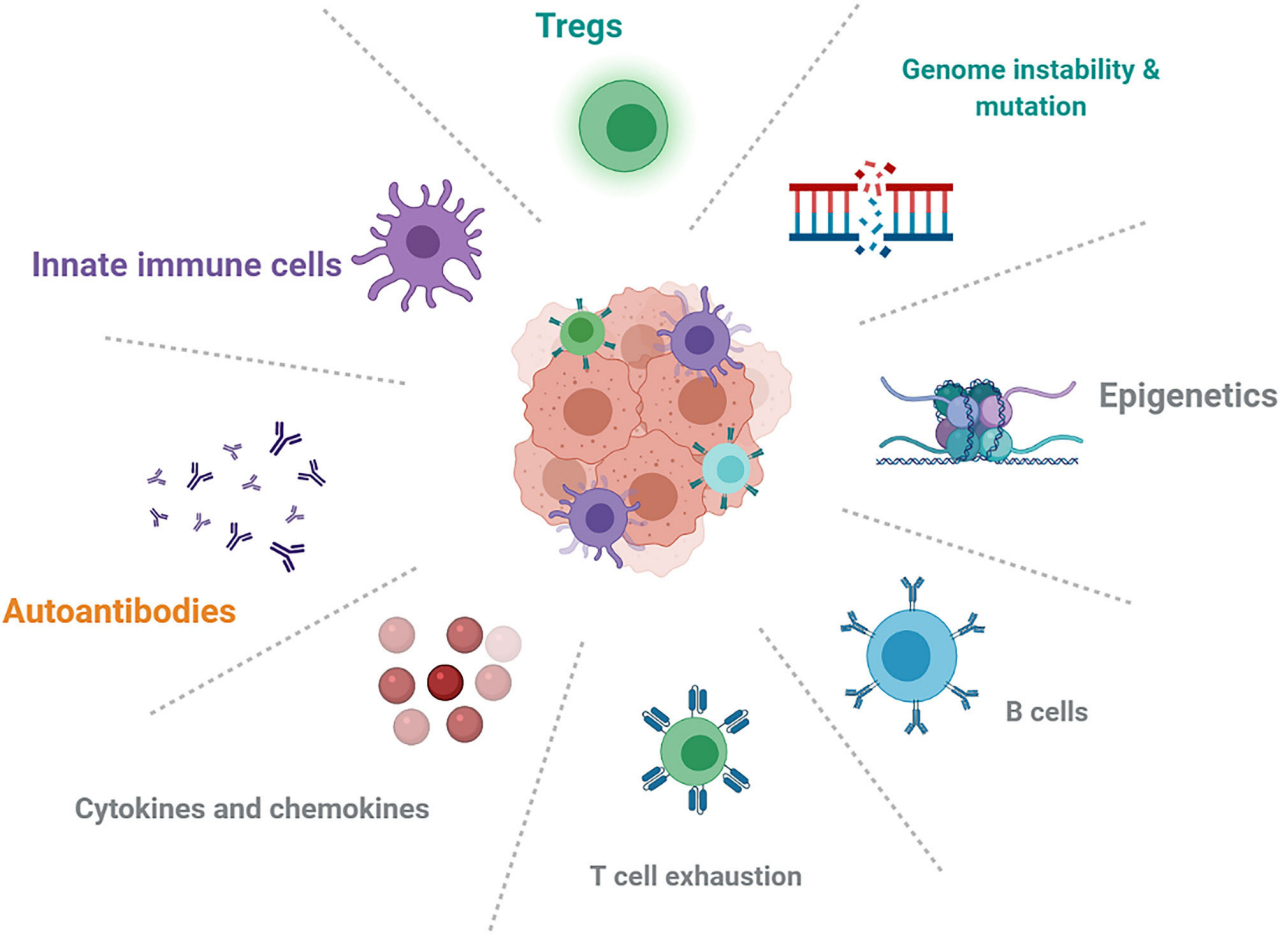
Future directions about the mechanistic understanding and possible biomarkers in autoimmune irAEs induced by ICIs.

The effects of genetics, epigenetics, pre-existing autoimmune conditions, and immune status (Figure 1) could play an important role in mechanistic understanding and predictive biomarkers. HLA class I homozygosity is related with reduced survival in patients with melanoma, possibly due to a T cell antigenic repertoire and reduced anti-tumor cytotoxicity (93). Early changes in B-cells could identify patients who are at increased risk of irAEs and indicated that strategies targeting B-cells might limit toxicities (19). IL-6 and chemokine CXCL-9 are important for the growth and development of tumors, but it is unclear whether baseline cytokine and chemokine levels are associated with overall cancer survival and the development of irAEs.

## Conclusion

While not among the most frequently encountered irAEs, rheumatic complications or diseases induced by ICIs are relatively common and are increasingly recognized, with musculoskeletal manifestations (including arthralgia, arthritis, myalgia, myositis) being the most common, and vasculitis, SS and SLE also detected in several cases. These complications can be clinically severe; therefore, early diagnosis and multidisciplinary management is of critical significance to attenuate pain and functional impairment, maintain immunotherapy, and preserve the quality of life of patients. Prospective large-scale studies will determine the clinical prevalence and characteristics of ICI-induced syndromes, find more predictive biomarkers, increase knowledge regarding diagnosis and management for physicians, and develop relevant therapeutic guidelines.

## Author Contributions

PS wrote the first draft of the manuscript. All authors participated in manuscript revision and have approved the submitted version of the manuscript.

## Conflict of Interest

The authors declare that the research was conducted in the absence of any commercial or financial relationships that could be construed as a potential conflict of interest.

## Publisher's Note

All claims expressed in this article are solely those of the authors and do not necessarily represent those of their affiliated organizations, or those of the publisher, the editors and the reviewers. Any product that may be evaluated in this article, or claim that may be made by its manufacturer, is not guaranteed or endorsed by the publisher.
